# The Activin Receptor, Activin-Like Kinase 4, Mediates *Toxoplasma Gondii* Activation of Hypoxia Inducible Factor-1

**DOI:** 10.3389/fcimb.2019.00036

**Published:** 2019-03-05

**Authors:** Agnieszka Lis, Mandi Wiley, Joan Vaughan, Peter C. Gray, Ira J. Blader

**Affiliations:** ^1^Department of Microbiology and Immunology, Jacobs School of Medicine and Biomedical Sciences, University at Buffalo, Buffalo, NY, United States; ^2^Department of Microbiology and Immunology, University of Oklahoma Health Sciences Center, Oklahoma City, OK, United States; ^3^Salk Institute, La Jolla, CA, United States

**Keywords:** toxoplasma and toxoplasmosis, hypoxia, transcripional regulation, parasite - host interactions, tgf-beta signaling

## Abstract

To grow and cause disease, intracellular pathogens modulate host cell processes. Identifying these processes as well as the mechanisms used by the pathogens to manipulate them is important for the development of more effective therapeutics. As an example, the intracellular parasite *Toxoplasma gondii* induces a wide variety of changes to its host cell, including altered membrane trafficking, cytoskeletal reorganization, and differential gene expression. Although several parasite molecules and their host targets have been identified that mediate- these changes, few are known to be required for parasite replication. One exception is the host cell transcription factor, hypoxia-inducible factor-1 (HIF-1), which is required for parasite replication in an oxygen-dependent manner. *Toxoplasma* activates HIF-1 by stabilizing the HIF-1α subunit, and this is dependent on the signaling from the Activin-Like Kinase (ALK) receptor superfamily. Here, we demonstrate that specific overexpression of the ALK family member, ALK4, increased HIF-1 activity in *Toxoplasma*-infected cells, and this increase required ALK4 kinase activity. Moreover, *Toxoplasma* stimulated ALK4 to dimerize with its co-receptor, ActRII, and also increased ALK4 kinase activity, thereby demonstrating that *Toxoplasma* activates the ALK4 receptor. ALK4 activation of HIF-1 was independent of canonical SMAD signaling but rather was dependent on the non-canonical Rho GTPase and JNK MAP kinase signaling pathways. Finally, *Toxoplasma* increased rates of ALK4 ubiquitination and turnover. These data provide the first evidence indicating that ALK4 signaling is a target for a microbial pathogen to manipulate its host cell.

## Introduction

When a host cell encounters an intracellular pathogen, DNA-binding transcription factors are activated either directly by pathogen-derived factors or indirectly by host-derived autocrine/paracrine-acting chemokines, cytokines, and growth factors. In general terms, the function of these host transcription factors can be categorized into three groups: (i) pro-host–protects the host from infection; (ii) pro-parasite–promotes pathogen growth; and (iii) bystander–genes that have no apparent impact on the host–pathogen interaction. STAT1 is a prototypical pro-host transcription factor since it regulates the expression of IFNγ-effector genes, which is required for resistance to *Toxoplasma* (Collazo et al., 2002; Lieberman et al., 2004). Although few pro-parasite transcription factors are known, the pathogen-derived factors that activate them, and the pathogen processes that rely on them are important to identify as they represent novel drug targets.

Hypoxia-inducible factor-1 (HIF-1) is a host cell transcription factor that is activated by a wide array of microbial pathogens (Nizet and Johnson, 2009). In mice infected with extracellular pathogens such as *Streptococcus pyogenes* and *Pseudomonas aeruginosa*, HIF-1 is important for host resistance by regulating neutrophil and macrophage phagocytic functions (Cramer et al., 2003; Peyssonnaux et al., 2005). HIF-1 is also activated by diverse classes of intracellular pathogens (Sodhi et al., 2000; Wakisaka et al., 2004; Arrais-Silva et al., 2005; Kempf et al., 2005; Peyssonnaux et al., 2005; Spear et al., 2006; Hartmann et al., 2008; Nakamura et al., 2009; Metheni et al., 2015; Fecher et al., 2016). In some of these infections, HIF-1 plays a key role in host defenses by regulating the expression of immunomodulatory molecules and metabolic enzymes. Less is known about whether HIF-1 can support the growth of intracellular pathogens. One notable exception is work from our laboratory demonstrating that HIF-1 is a key pro-parasite transcription factor in cells infected with the protozoan parasite *Toxoplasma gondii* (Spear et al., 2006).

HIF-1 is a heterodimer composed of α and β subunits that is activated when O_2_-dependent degradation of the HIF-1α subunit is prevented due to hypoxic stress. However, *Toxoplasma* does not activate HIF-1 merely by consuming O_2_ and triggering localized hypoxic responses. Rather, the parasite activates HIF-1 by down regulating the prolyl hydroxylase 2 (PHD2) enzyme (Wiley et al., 2010) whose hydroxylation of HIF-1α targets it for proteasomal degradation. Using pharmacological, cellular, and genetic inhibitors, we demonstrated that signaling from the Activin-Like Kinase receptor superfamily (ALK4,5,7) is required for HIF-1 activation in *Toxoplasma*-infected cells (Wiley et al., 2010; Brown et al., 2014).

The ALK4,5,7 receptors are a family of conserved serine/threonine kinase receptors that bind about 30 different ligands. These ligands include Activin A (ALK4), TGFβ (ALK5), and Nodal (ALK7) (Miyazono et al., 2001). ALK4,5,7 receptors do not bind ligands on their own but do so only after their ligand binds a second serine/threonine kinase type II receptor [TGFBR2 (ALK5) and ActRII (ALK4 and ALK7)], which induces the homodimers of each receptor type to multimerize (Attisano and Wrana, 2002). Receptor multimerization allows the cytoplasmic domain of the type II receptor to phosphorylate ALK4,5,7, which leads to activation of the type I receptor's kinase activity. Substrates of ALK4,5,7 kinases mediate most of the physiological changes induced by the ligand, and the SMAD2/SMAD3 transcriptional regulators are the best characterized effectors and are therefore considered the canonical signaling pathway (Attisano and Wrana, 2002). Other non-canonical pathways are also activated by ALK4,5,7; and these include MAP and PI-3 kinases (Zhang, 2009) although the mechanisms by which they are activated is less clear. Here, we demonstrate that *Toxoplasma* activates ALK4 to trigger HIF-1 activity. We also demonstrate that HIF-1 activation by ALK4 is independent of SMAD2/3 but rather requires host Rho GTPase and JNK MAP kinase signaling.

## Materials and Methods

### Cells and Parasites

The RH *Toxoplasma* strain (from ATCC; Manassas, VA) and the GRA24 knockout (from Dr. Mohamed Ali Hakimi (CNRS; Grenoble, France) was passaged in human foreskin fibroblasts (HFFs) and murine embryonic fibroblasts (MEFs) in Dulbecco's Minimal Essential Medium (DMEM) supplemented with 10% heat-inactivated fetal bovine serum, glutamine and penicillin (100 U/mL)/streptomycin (10 mg/mL) as described (Wiley et al., 2010). All cells and parasites were routinely tested for Mycoplasma contamination (MycoAlert from Lonza; Basel, Switzerland) and found to be negative. Unless otherwise noted, the highest grade possible of chemicals were purchased from Sigma (St. Louis, MO). In addition, experiments were performed under normoxic conditions unless otherwise noted. Cells were grown under low O_2_ conditions using an INVIVO_2_ Hypoxia Chamber (Baker Instruments; Sanford, ME).

### Luciferase Assay

Luciferase assays were performed as previously described (Wiley et al., 2010). Briefly, cells were transfected using Lipofectamine 2,000 (Invitrogen; Carlsbad, CA) in 24-well plate with the indicated plasmids (400 ng total) and grown for 24 h at 37°C. The cells were then mock or parasite infected at a MOI of 4 and incubated for 18 h. The cells were harvested and luciferase activity measured using the Dual Glo Luciferase Reporter Assay (Promega; Madison, WI). U0126 was dissolved in DMSO. Recombinant Lethal Factor/Protective Antigen (LF; kindly provided by Dr. Jimmy Ballard from the University of Oklahoma Health Sciences Center) was used as previously described (Phelps et al., 2008). The plasmids used for this study are described in Supplemental Table 1.

### Western Blotting

Host cells were mock or parasite infected (MOI of 4) for the indicated times, washed 3 times with ice-cold PBS, and lysed on ice with lysis buffer (50 mM TRIS-HCl pH 7.4, 1% NP-40, 150 mM NaCl, 0.1% SDS, 0.25% Sodium Deoxycholate, 10 mM NaF, 20 mM Na_3_VO_4_, 10 mM EDTA, 100 mM beta-glycerophosphate, and 1 X protease inhibitor cocktail (Roche; Indianapolis, IN). Lysates were collected, centrifuged at 16,000 xg to remove cell debris, and protein concentrations determined. Equal amounts of proteins were separated by SDS-PAGE, transferred to nitrocellulose membrane, blocked for 1 h with LI-COR blocking solution (LICOR; Lincoln, NE), incubated overnight at 4°C with primary antibodies (see Supplemental Table 2) in 5% bovine serum albumin in TTBS (50 mM Tris pH 7.4, 150 mM NaCl, 0.1% Tween-20), followed by a 2-h incubation with Alexa Fluor 680- or 800-conjugated secondary antibodies (Li-COR). Blots were visualized using the LI-COR Odyssey scanner and quantified using the Imager's software.

### Immunoprecipitation

Mock- or parasite-infected host cells were lysed on ice with immunoprecipitation buffer (50 mM Tris-HCl buffer pH 7.5, 150 mM NaCl, 0.5% NP-40, 5 mM EDTA, 10% glycerol, 10 mM NaF, 20 mM Na_3_VO_4_ plus 1 X protease inhibitor cocktail). Lysates were clarified and equal amounts of protein were incubated overnight with primary antibodies (see Supplemental Table 2) at 4°C, and then incubated for an additional 2–3 h with protein G agarose (ThermoFisher; Waltham, MA) at 4°C. Beads were washed 5 times with lysis buffer and eluted with SDS-PAGE sample buffer for 5 min at 95°C and analyzed by Western blotting.

### *In vitro* Kinase Assay

Host cells were incubated overnight with DMEM containing 0.2% heat-inactivated serum and then mock or parasite-infected (MOI = 4) for the indicated times. Cells were then lysed in modified RIPA buffer (25 mM Tris-HCl pH 7.4, 0.1% SDS, 1% NP-40, 150 mM NaCl, 1 mM NaF, 10 mM Na_3_VO_4_, 25 mM beta-glycerophosphate, 0.25% sodium deoxycholate, and 1 X protease inhibitor cocktail), and 500 μg of the lysates were incubated overnight with anti-ALK4-purified rabbit antibody (Lebrun and Vale, 1997) at 4°C. Protein A agarose beads were added for 3 h, collected by centrifugation, washed 3 times with modified RIPA buffer and 2 times with kinase assay buffer (25 mM HEPES pH 7.6, 1 mM DTT, 10 mM MgCl_2_, 25 mM beta-glycerophosphate, 200 μM ATP). Kinase reactions were started by adding 0.8 μg of SMAD2–GST (Sigma) and 5 μCi^32^P-ATP (6,000 Ci/mmole; Perkin Elmer; Waltham, MA) to each tube. After 2 h, the reactions were extensively washed with kinase assay buffer, separated by SDS-PAGE, and the gels were dried and subjected to autoradiography.

### Immunofluorescence

HFFs plated on glass coverslips were treated with either 50 ng/mL of Activin A or parasite-infected (MOI of 4) for 2 h and then fixed with 2% paraformaldehyde (in phosphate buffered saline (PBS) for 20′ at room temperature and then washed 3 times with ice-cold PBS. Cells were permeabilized with 0.1% Triton X-100 for 30′, blocked overnight with 3% bovine serum albumin in PBS at 4°C. Then, they were incubated with primary antibodies (Tables) for 2 h, washed 3 times in PBS and incubated with Alexa 488- or Alexa 594-conjugated secondary antibodies for 1 h. Coverslips were washed and then mounted on slides in Vectashield-containing DAPI (Vector Labs, Burlingame, CA) mounting medium. Coverslips were imaged by fluorescence microscopy and images acquired using a 100 X Plan Apo oil immersion 1.46 527 numerical aperture lens on a motorized Zeiss Axioimager M2 microscope equipped with 528 an Orca ER charge-coupled-device (CCD) camera (Hamamatsu, Bridgewater, NJ).

## Results

### ALK4 Potentiates HIF-1 Activation in *Toxoplasma*-Infected Cells

**Figure 1:**
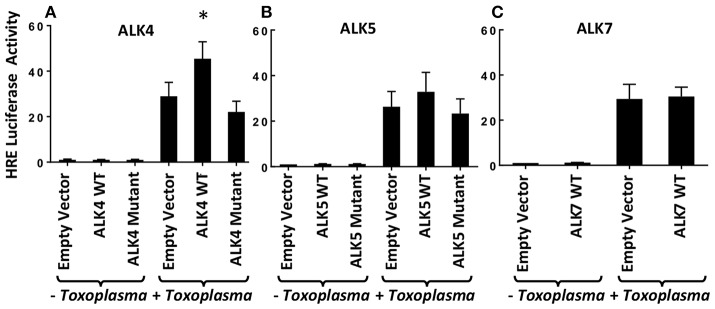
ALK4 Overexpression Potentiates HIF-1 Activity in Toxoplasma-Infected Cells. MEFs were transfected overnight with pHRE-luc together with wild-type or kinase-deficient ALK4 **(A)**, ALK5 **(B)**, or ALK7 **(C)** (or empty vector as a control). The cells were mock or parasite infected for 18 h and luciferase activity was measured. Shown are the averages ± standard errors of at least 3 independent experiments. ^*^<*p*< 0.05 (unpaired Student's *t*-test).

Previously, we demonstrated that *Toxoplasma* signals through the ALK4,5,7 receptor superfamily to activate HIF-1 (Wiley et al., 2010). To determine which receptor(s) is involved in HIF-1 activation, we hypothesized that overexpression of a relevant receptor would potentiate HIF-1 activity in *Toxoplasma*-infected cells. Thus, cells were transfected with plasmids expressing a HIF-1–regulated firefly luciferase reporter (HRE-luc), a constitutively expressed Renilla luciferase reporter (to normalize firefly luciferase activity), and either ALK4, ALK5, or ALK7 (or an empty vector as a control). The transfected cells were infected with *Toxoplasma*, and 18 h later the cells were lysed and their luciferase activity was measured. We found that only ALK4 overexpression significantly increased HRE-luc activity in *Toxoplasma*-infected cells (Figure 1). Increases in luciferase activity required ALK4 kinase activity since an ALK4 kinase dead mutant was unable to increase luciferase activity following *Toxoplasma* infection. We did not, however, note a dominant negative effect of the mutant on HIF-1 activation indicating that either expression levels were not high enough or that *Toxoplasma* and ALK4 interact trough novel mechanism.

### *Toxoplasma* Activates ALK4

**Figure 2:**
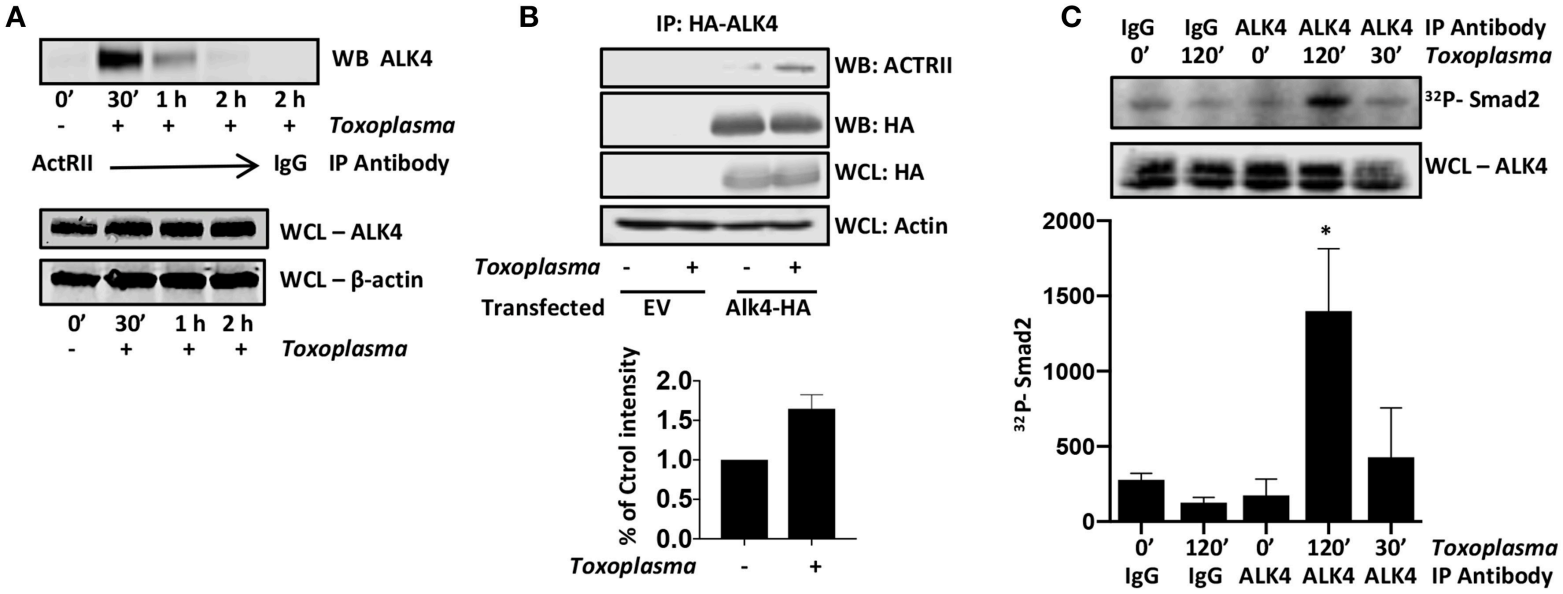
Toxoplasma Induces ALK4/ActRII Dimerization and Activation. **(A)** ActRII was immunoprecipitated from lysates prepared from mock- or parasite-infected cells and immune complexes were Western blotted to detect ALK4. ALK4 and β-actin (as a loading control) levels were assessed in whole cell lysates (WCL). **(B)** Host cells transfected with plasmid encoding HA-tagged ALK4 (or an empty vector) was mock- or parasite-infected for 30′. Lysates were prepared and incubated with anti-HA antibody and Protein A Sepharose. Immune complexes were collected and Western blotted using anti-ActRII antisera. Whole cell lysates (WCL) were also Western blotted to assess total protein levels. **(C)** Lysates from mock- or parasite-infected cells were incubated with the indicated antibody and Protein A Sepharose. Immune complexes were collected and then incubated with recombinant SMAD2 and 32Pγ-ATP. Reactions were separated by SDS-PAGE and then the gels were dried, exposed to a Phosphorimager screen, and analyzed by densitometry. Shown is a representative autoradiograph and the graph represents the average and standard deviation of three independent experiments. *<*p*< 0.05 (One Way ANOVA).

ALK4 activation is a multistep process in which ALK4 homodimers interact with homodimers of a type II ActRII receptor only after the type II receptors become ligand bound. Type II receptors are constitutively active serine/threonine kinases and ligand-binding allows to bind and phosphorylate ALK4, leading to an increase in the kinase activity of ALK4. To test whether *Toxoplasma* induces ALK4/ActRII multimerization, cells were mock- or parasite-infected for increasing amounts of time and then immunoprecipitated with anti-ActRII antisera or IgG as a control. The immunoprecipitates were collected and Western blotted to detect ALK4. ALK4/ActRII interactions increased within 30′ post infection and then decreased by 2 hpi (Figure 2A and Supplemental Figure 1). To confirm *Toxoplasma* ALK4/ActRII interactions, cells were transfected with either an empty vector or a HA-tagged ALK4 expression construct and were mock- or parasite-infected 30′. HA-tagged ALK4 was immunoprecipitated with anti HA antibody and immune complexes blotted to detect ActRII. Consistent with the ActRII immunoprecipitation data, *Toxoplasma* increased the abundance of ActRII in the ALK4 immunoprecipitates (Figure 2B).

ALK4 kinase activity is stimulated after it multimerizes with ActRII. We therefore immunoprecipitated ALK4 from the mock- and parasite-infected cells, and the immunoprecipitates were incubated in kinase assay buffer with γ-^32^P-ATP and recombinant SMAD2, which is an ALK4 substrate (Heldin et al., 1997). The reactions were separated by SDS-PAGE, and radiolabeled SMAD2 was detected by Phosphorimager analysis. We found that infection significantly increased ALK4 kinase activity 2 hpi (Figure 2C), suggesting that ALK4 remains active even after ActRII and ALK4 dissociate from one another. These data indicate that *Toxoplasma* activates ALK4 and uses this receptor to activate HIF-1.

### *Toxoplasma* Activates HIF-1 Independently of Canonical SMAD Signaling

**Figure 3:**
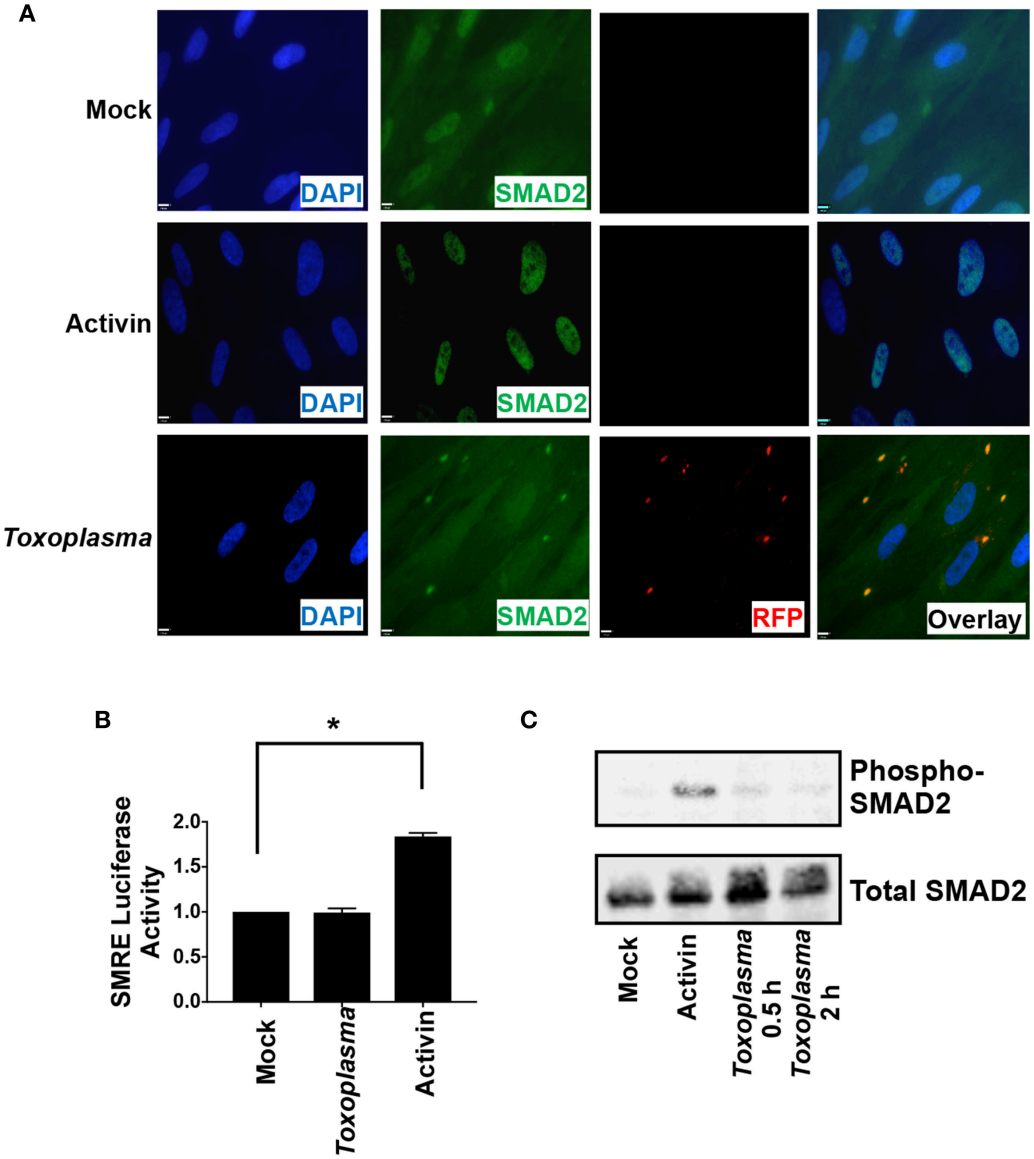
HIF-1 Activation is Independent of SMAD2/3 Signaling. **(A)** HFFs plated on coverslips were mock infected, infected with RH-RFP parasites for 2 h, or treated with Activin A for 2 h. Cells were then fixed and SMAD2 detected by immunocytochemistry. Coverslips were then mounted and cells visualized by immunofluorescence microscopy. Note the apparent cross reactivity between *Toxoplasma* and the anti-SMAD2 antibody. **(B)** pSMRE-luc transfected cells were treated Activin A (50 ng/ml) or mock- or parasite-infected. Lysates were collected 18 h later and luciferase activity was measured. Shown are averages and standard deviations of 3 independent assays performed in triplicate *<*p*< 0.001 (One Way ANOVA) **(C)** Lysates from mock-infected, parasite-infected, and Activin-treated cells were Western blotted with antibodies that detect phosphorylated or total SMAD2.

ALK downstream signaling comprises the canonical SMAD2/3 pathway and non-canonical pathways that includes MAP kinases. SMAD2 and SMAD3 are resident cytoplasmic DNA-binding proteins that are phosphorylated by activated ALK4 receptors. Once phosphorylated, SMAD2/3 binds SMAD4 and the complex traffics to the nucleus, where it binds DNA and regulates transcription (Macías-Silva et al., 1996). To assess SMAD2/3 activity in *Toxoplasma*-infected cells, SMAD2 subcellular localization was compared among mock- and *Toxoplasma*-infected cells (2 hpi) and activin-treated cells as a control. SMAD2 accumulated in nuclei of activin-treated cells but not in *Toxoplasma*-infected cells (Figure 3A). We did note that SMAD2 levels were apparently slightly lower in nuclei of *Toxoplasma*-infected cells but were precluded from drawing any conclusions since the SMAD2 antisera cross reacts with tachyzoites.

We next examined SMAD2/3 activity in *Toxoplasma*-infected cells using a SMAD2/3-dependent luciferase (SMRE-luc) reporter plasmid. Thus, pSMRE-luc-transfected host cells were either mock- or parasite-infected or treated with activin as a positive control. The cells were lysed 18 h later and luciferase activity was measured. We found that while activin increased luciferase activity *Toxoplasma* infection did not (Figure 3B). We next tested whether *Toxoplasma* infection stimulates SMAD2/3 phosphorylation by Western blotting lysates from mock- and parasite-infected cells using antibodies that recognize either total or phosphorylated SMAD2. In contrast to activin, which robustly increased SMAD2 phosphorylation, infection with *Toxoplasma* was unable to stimulate phosphorylation (Figure 3C). Together, these data indicate that while *Toxoplasma* engages and activates ALK4 it does so in a manner that prevents it from activating canonical SMAD2/3 signaling.

### Host JNK MAP Kinase Signaling Is Necessary for *Toxoplasma* Activation of HIF-1

**Figure 4:**
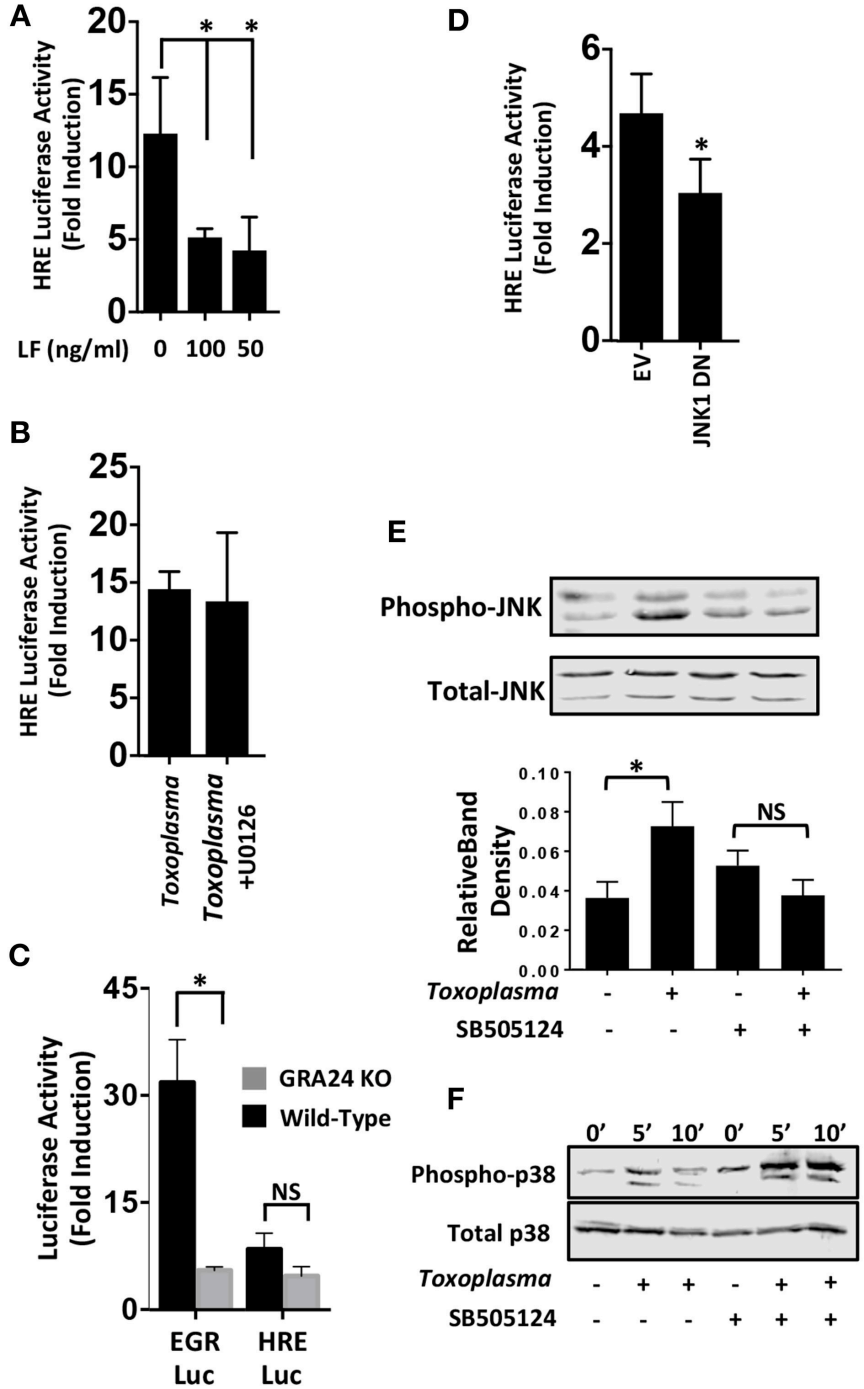
JNK Signaling Is Important for HIF-1 Activation in *Toxoplasma*-infected Cells. **(A)** pHRE-luc-transfected cells were mock treated or treated with *B. anthracis* Lethal Factor (LF) for 6 h and then infected with *Toxoplasma*. Lysates were collected 18 h later and luciferase activities were measured. Shown are the average infection-induced fold inductions and standard deviations of 3 independent experiments. * < *p* < 0.05 (One Way ANOVA). **(B)** Mock- or *Toxoplasma*-infected pHRE-luc-transfected cells were treated with either DMSO or the ERK inhibitor U0126 (10 μM). Lysates were collected 18 h later and luciferase activities measured. Shown are average infection-induced fold inductions ± standard deviations of 3 independent experiments. **(C)** pHRE-luc or pEGR-luc transfected cells were mock-infected or infected with wild-type RH or GRA24 knockout parasites. Lysates were collected 18 h later and luciferase activities measured. Shown are the average infection-induced fold inductions ± standard deviations of 3 independent experiments. NS *p* > 0.05 (Student's *t*-test). *p < 0.05 (Student's *t*-test). **(D)** pHRE-luc-transfected cells were co-transfected with either empty vector or JNK1 dominant negative (DN) and then mock or parasite infected. Lysates were collected 18 h later, and luciferase activities was measured. Shown are average infection-induced fold inductions ± standard deviations of 3 independent experiments.*p< 0.05 (Student's *t*-test). **(E)** Host cells treated with either DMSO or SB505124 (5 μM) for 2 h and then mock or parasite infected. Lysates were collected 2 h later and Western blotted to detect total or phosphorylated JNK1/2. Graph shows averages of phosphorylated:total JNK1/2 ratios ± standard deviations from 3 independent experiments. NS *p*>0.05 (Student's *t*-test).*p< 0.05 (Student's *t*-test). **(F)** Host cells were pretreated for 2 h with SB505124 (5 μM) and then mock or parasite infected for the indicated times. Lysates were prepared and Western blotted to detect total or phosphorylated p38 MAPK.

We next examined MAP kinases (MAPKs) because they; (i) are ALK4,5,7-regulated non-canonical signaling pathways (Zhang, 2009), (ii) regulate HIF-1(Comerford et al., 2002; Semenza, 2002), and (iii) are activated by *Toxoplasma* (Derynck and Zhang, 2003; Kietzmann et al., 2016). To test whether MAPK signaling was involved in HIF-1 activation in *Toxoplasma*-infected cells, HRE-luc–expressing cells were mock-treated or treated with *Bacillus anthracis* Lethal Factor (LF), which inactivates MAPK signaling by cleaving MAP kinase kinases (Bardwell et al., 2004). We found that luciferase activity was significantly reduced by LF, indicating the involvement of a MAPK signaling module in *Toxoplasma* activation of HIF-1 (Figure 4A).

Mammalian cells express three major MAPK signaling cascades (ERK, p38 and JNK). Because pharmacological inhibitors against p38 and JNK MAPKs block parasite growth (Wei et al., 2002; Dittmar et al., 2016), we first tested the effect of the ERK inhibitor, U0126 (10 μM), which does not affect parasite growth (Dittmar et al., 2016), We found that HIF-1 activation was unaffected by treatment with this compound (Figure 4B) which is consistent with the finding that this compound has no effect on parasite growth at either 21 or 3% O_2_ [(Dittmar et al., 2016) and data not shown]. p38 MAPK activation in *Toxoplasma*-infected cells is dependent on a dense granule protein named GRA24 (Braun et al., 2013). We therefore compared the ability of wild-type or GRA24 knockout parasites to activate HIF-1 or EGR2, which is a host cell transcription factor whose activation by *Toxoplasma* is dependent on GRA24/p38 MAPK signaling (Braun et al., 2013; Dittmar et al., 2016). While EGR2 activation was reduced by the loss of GRA24, HIF-1 activation was comparable between the wild-type and GRA24 knockout parasites (Figure 4C).

To examine the role of JNK signaling in HIF-1 activation, cells were transfected with the HIF-1 luciferase reporter along with a plasmid encoding a dominant negative JNK mutant. Cells were infected and after 18 h luciferase activity was measured and found to be significantly reduced in the *Toxoplasma*-infected cells expressing the JNK mutant (Figure 4D). JNK is activated in *Toxoplasma*-infected cells (Valère et al., 2003; Morgado et al., 2011), and we therefore tested whether JNK activation was ALK4 dependent. We previously showed that SB505124, an ALK4,5,7 inhibitor, blocks HIF-1 activation. It also prevents *Toxoplasma* replication by inhibiting a parasite MAPK homolog, but this effect is only apparent at later times post infection (DaCosta Byfield et al., 2004; Wiley et al., 2010; Brown et al., 2014). Thus, we pretreated cells with SB505124 or DMSO vehicle (as a control) for 30′ and then mock- or parasite-infected them for 2 h. Cell lysates were Western blotted to detect phospho-specific or total JNK. *Toxoplasma*-induced JNK1 phosphorylation was reduced by SB505124 (Figure 4E). In contrast, SB505124 did not inhibit *Toxoplasma* induction of p38 MAPK phosphorylation and appeared to potentiate its phosphorylation. These data indicate that that the drug does not unexpectedly inhibit other host cell signaling pathways impacted by *Toxoplasma* infection (Figure 4F).

### Host Rho GTPase Signaling Is Required for HIF-1 Activation

**Figure 5:**
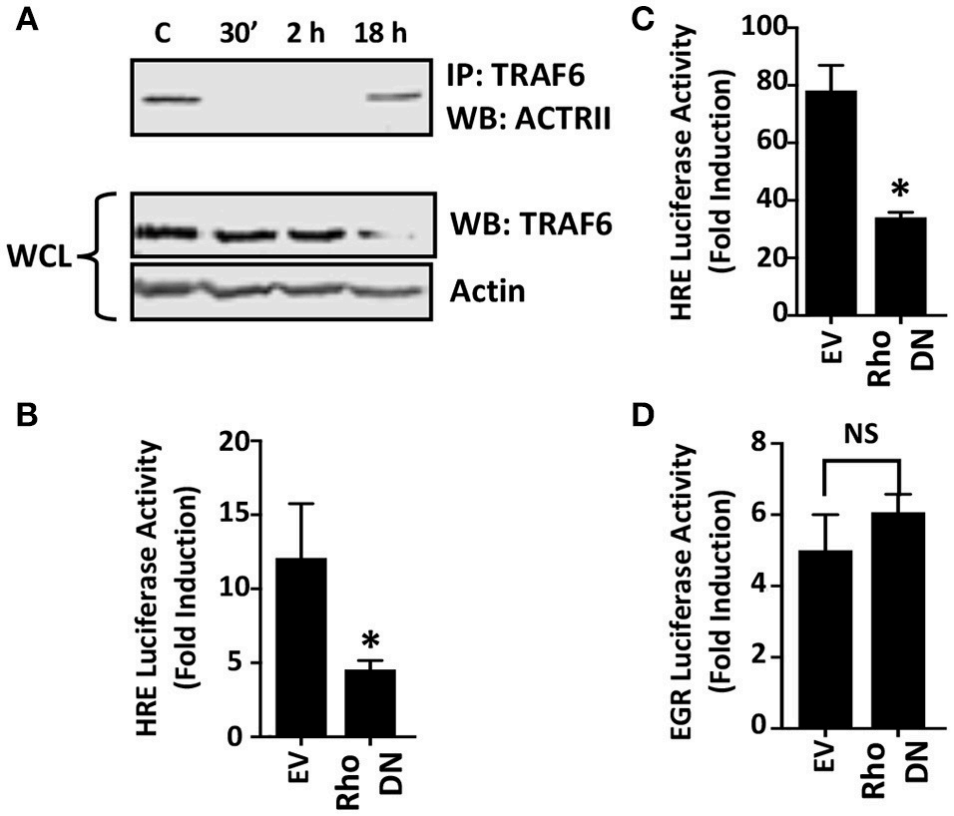
Rho GTPase Signaling is Important for HIF-1 Activation in *Toxoplasma*-infected Cells. **(A)** Host cells were mock-infected or infected with tachyzoites for 30′, 2 or 18 h. TRAF6 was then immunoprecipitated and ACTRII was detected by Western blotting in either the immune complexes or whole cell lysates (WCL) (actin was used as a loading control). **(B,C)** pHRE-luc-transfected cells were co-transfected with either an empty vector or a RhoA dominant negative (DN) mutant expression construct and then mock- or parasite-infected **(B)** or exposed to 3% O_2_ **(C)** Lysates were collected 18 h later and luciferase activity was measured. Shown are the average fold inductions (infection or low O_2_ stimulated) ± standard deviations of 3 independent experiments. **(D)** pEGR-luc-transfected cells were co-transfected with empty vector or RhoA dominant negative (DN) mutant and then mock or parasite infected. Lysates were collected 18 h later and luciferase activities measured. Shown are the average fold inductions +/- standard deviations of 3 independent experiments. *p < 0.05 (Student's *t*-test).

Two distinct pathways mediate TGFβ activation of JNK. First, the JNK-activating MAPK kinase, TAK1, is activated via recruitment of the TRAF6 ubiquitin ligase to the ALK5/TGFβRII receptor complex (Yamashita et al., 2008). To test whether *Toxoplasma* increases TRAF6 association with ALK4/ActRII, cells were mock- or parasite-infected for 2 h. Then TRAF6 was immunoprecipitated and the immune complexes were Western blotted to detect ActRII. In contrast to TGFβ (Yamashita et al., 2008), TRAF6 recruitment to the ALK4/ActRII complex was significantly decreased early after infection although it was restored at 18 hpi (Figure 5A). Since receptor binding is required for TRAF6 activity, these data therefore indicate that TRAF6 is most likely not involved in ActRII/ALK4-mediated activation of HIF-1 in *Toxoplasma*-infected cells.

Rho GTPase signaling is a second pathway that mediates TGFβ activation of JNK (Atfi et al., 1997). To test whether this GTPase is important for HIF-1 activation in *Toxoplasma*-infected cells, HRE-luciferase reporter transfected host cells were co-transfected with either a plasmid that expresses a dominant negative Rho GTPase mutant or an empty vector as a control. The cells were then mock- or parasite-infected for 18 h at which time luciferase activity was measured. The data indicated that HIF-1–dependent luciferase activity was significantly reduced in *Toxoplasma*-infected cells expressing the dominant negative Rho GTPase mutant (Figure 5B). As a positive control, we demonstrated that the dominant negative Rho mutant reduced hypoxic activation of the HRE-luc reporter, a finding consistent with previous reports that Rho signaling is important for HIF-1 activation (Hayashi et al., 2005) (Figure 5C). In contrast, expression of the Rho GTPase mutant had no apparent effect on parasite activation of the EGR-luciferase reporter (Figure 5D) indicating that expression of the mutant did not have unexpected effects on host cell signaling.

### *Toxoplasma* Induces ALK4 Turnover

**Figure 6:**
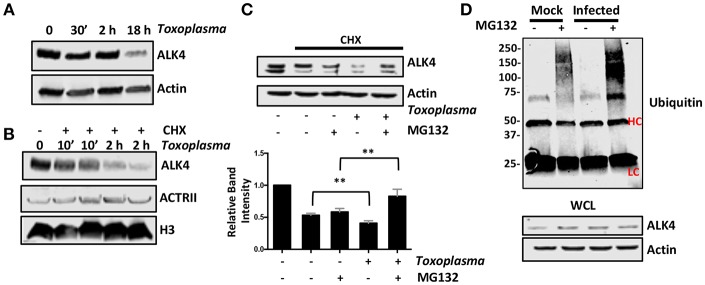
*Toxoplasma* Infection Increases Rates of ALK4 Turnover. **(A)** Lysates from mock- or parasite-infected cells were Western blotted to assess ALK4 or actin (as a loading control) protein levels. **(B)** Host cells were pretreated with cycloheximide (CHX, 50 μg/ml) for 30′ and then infected with *Toxoplasma* for 10′ or 2 h. The cells were lysed and Western blotted to detect ALK4 and ACTRII. Histone H3 was used as a loading control. **(C)** Host cells were pretreated as indicated with CHX (50 μg/ml) and MG132 (10 μM) for 30′ and then mock- or parasite-infected for 2 h. Lysates were prepared 2 h later and Western blotted to detect ALK4 or actin as a loading control. Graph represents average relative band intensities (ALK4/actin) +/- standard deviations of 3 independent experiments. **p < 0.01 (One Way ANOVA). **(D)** Cells were pretreated as indicated with MG132 (10 μM) for 30′ and then mock or parasite-infected for 2 h. Lysates were prepared and ALK4 immunoprecipitated using ALK4 antisera. Immune complexes were separated by SDS-PAGE and Western blotted to detect ubiquitin. Note increased high molecular weight staining in the *Toxoplasma*-infected samples. HC and LC highlights antibody heavy and light chains, respectively. Shown is a representative image from 3 independent experiments.

Once activated, ALK4,5,7 receptors are endocytosed and degraded by both proteasome-dependent and -independent mechanisms (Kavsak et al., 2000; Di Guglielmo et al., 2003). To test whether *Toxoplasma* induces a similar fate for ALK4, we first assessed its steady state levels after 0.5, 2, and 18 h post infection and found that ALK4 levels were significantly reduced at 18 hpi (Figure 6A). Since infection does not reduce ALK4 mRNA levels [(Blader et al., 2001; Kim et al., 2007; Saeij et al., 2007) and data not shown], we tested whether *Toxoplasma* decreased ALK4 half-life. Thus, cells were pretreated for 30′ with the protein synthesis inhibitor, cycloheximide (CHX), and then mock- or parasite-infected for 10′ or 2 h. Lysates were prepared and Western blotted to detect ALK4, ActRII, or histone H3 as a loading control. In mock-infected cells, CHX reduced ALK4 levels as early as 10 min and ALK4 levels were significantly reduced by 2 h. While infection did not have an apparent affect on ALK4 turnover after 10′, it did so at 2 hpi (Figure 6B). In contrast, turnover rates of either ActRII isoforms were unaffected by infection.

Next, we tested whether ALK4 turnover was proteasome-dependent by examining ALK4 levels in CHX-pretreated cells that were mock- or parasite-infected in the absence or presence the proteasome inhibitor, MG132. We found that MG132 led to increased ALK4 levels in the *Toxoplasma*- but not mock-infected cells (Figure 6C). Finally, ALK4 was immunoprecipated from mock- or parasite-infected cells (2 hpi) that were treated with MG132 and then ubiquitin was detected in the immune complexes by Western blotting. The data indicated that infection led to increased ubiquitin staining in the MG132-treated *Toxoplasma*-infected cells (Figure 6D). Taken together, these data indicate that infection increases ubiquitination and proteasomal degradation of ALK4.

## Discussion

Intracellular pathogens create their replicative niches by secreting factors that interact with host cell proteins. Many of these host cell proteins are intracellular and are modulated by pathogen-derived factors that are exposed to the host cytoplasm. Others are cell surface receptors that are engaged either by the pathogen before they infect a host cell or by a host-derived factor whose release is stimulated by exposure to the pathogen. Here, we extend on our previous work (Wiley et al., 2010) and report that ALK4 is a host cell surface receptor kinase that is activated by *Toxoplasma* and is important for activating HIF-1. Our work, however, does not resolve whether ALK4 is necessary and sufficient for this process since ALK4 deficient cells are unavailable. In addition, ALK4 siRNAs, which had no dramatic effect on *Toxoplasma* activation of HIF-1 (not shown) only reduced target protein abundance by only ~70%. Thus, we cannot exclude possibilities that other receptors can compensate for decreased ALK4 activation or that limited ALK4 expression remains sufficient to mediate HIF-1 activation.

In *Toxoplasma*-infected cells, ALK4 only appears to regulate JNK without triggering other known downstream signaling pathways such as the SMADs. The mechanism(s) underlying this unique signaling specificity remains unknown. One possibility is that besides regulating receptor abundance, ubiquitin dependent degradation of ALK4,5,7 creates a signaling platform that promotes specific activation of JNK while blocking canonical SMAD2/3 signaling (Zuo and Chen, 2009). In addition, other host cell signaling pathways may be more directly and robustly regulated by *Toxoplasma* effector proteins that are introduced into the host cell during infection (e.g., GRA24/p38 MAPK, Braun et al., 2013) and therefore sequestered from interacting with ALK4.

Consistent with earlier work that *Toxoplasma* activates host Rho GTPases (Na et al., 2013), we found that Rho GTPase signaling was important for activating HIF-1 although it remains unclear how it integrates with JNK signaling. One possibility is that a Rho-GTP effector protein may directly signal to activate JNK or an upstream MAPK kinase. Alternatively, Rho may regulate JNK indirectly by affecting ALK4 endocytosis/intracellular trafficking. This may occur by virtue of Rho regulating actin stress fiber formation, which is necessary to coordinate caveolin-associated membrane domains (Stahlhut and van Deurs, 2000; Prieto-Sánchez et al., 2006). However, *Toxoplasma* does not appear to induce host cell stress fiber assembly (Morisaki et al., 1995; Coppens et al., 2006), and the rates of endocytosis/pinocytosis are not increased by infection (Jones and Len, 1976; Sweeney et al., 2010). Thus, we hypothesize that *Toxoplasma* utilizes Rho signaling to activate JNK through a more direct approach.

*Toxoplasma* activates HIF-1 via a parasite-derived secreted factor (Spear et al., 2006) that either directly engages ALK4 or stimulates the expression and release of a host factor that does so. In this manner, *Toxoplasma* can activate ALK4 in either the cells that it is infecting or neighboring uninfected cells. This suggests that besides the infected cell *Toxoplasma* may be modulating its microenvironment to establish favorable growth conditions. Besides HIF-1, the parasite also impacts its microenvironment by modulating cell cycle progression of neighboring uninfected cells (Lavine and Arrizabalaga, 2009). In addition, *Toxoplasma*-derived micronemal proteins that are shed from the parasite during invasion and therefore could interact with neighboring uninfected cells (Muniz-Feliciano et al., 2013) activate host Epithelial Growth Factor Receptor. Thus, we hypothesize that activation of HIF-1 and these other pathways may not only impact the infected cell but neighboring cells that are awaiting infection as well as other cells (e.g., monocytes and T-cells) recruited to the site of the infection. This may be potentially important as ALK4 signaling has been demonstrated to regulate regulatory T-cell development (Huber et al., 2009; Semitekolou et al., 2009; Tousa et al., 2017) as well as macrophage activation, polarization, and function (Ogawa et al., 2006; Zhou et al., 2009; Sierra-Filardi et al., 2011). Our future work will explore the global impact that the ALK4/HIF-1 signaling may have in the fate of *Toxoplasma* infections.

## Author Contributions

AL designed and performed experiments, interpreted results, and wrote the manuscript. MW designed and performed experiments, interpreted results, and edited the manuscript. JV and PG generated reagents, interpreted results, and edited the manuscript. IB designed experiments, interpreted results, and wrote the manuscript.

### Conflict of Interest Statement

The authors declare that the research was conducted in the absence of any commercial or financial relationships that could be construed as a potential conflict of interest.

## Supplementary Material

Supplemental Figure 1 *Toxoplasma* Induces ALK4/ActRII Dimerization and Activation. ActRII was immunoprecipitated from lysates prepared from mock- or parasite-infected cells (30′ post-infection) using anti ActRII or IgG as a control). Immune complexes were Western blotted to detect ALK4. ALK4 levels were assessed in whole cell lysates (WCL).

Supplemental Table 1 List of plasmids used in the study.

Supplemental Table 2 List of antibodies used in the study.
