# Supplementary figures and images for: The Activin Receptor, Activin-Like Kinase 4, Mediates Toxoplasma Gondii Activation of Hypoxia Inducible Factor-1
Source: Front Cell Infect Microbiol. 2019 Mar 5;9:36. doi: 10.3389/fcimb.2019.00036 (PMC6411701; doi:10.3389/fcimb.2019.00036)

## Slide 1
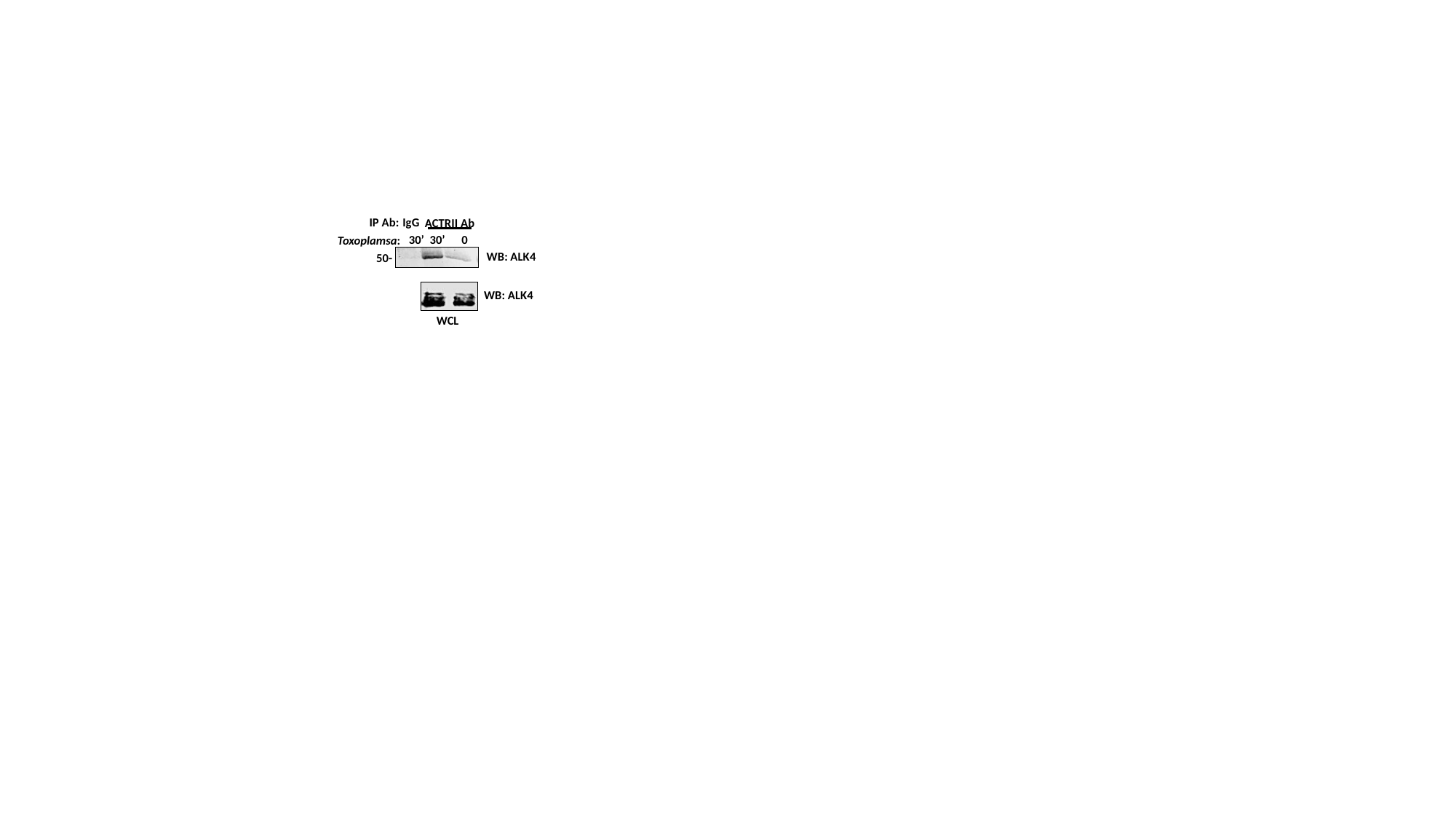

IgG
IP Ab:
ACTRII Ab
 30’ 30’ 0
Toxoplamsa:
WB: ALK4
50-
WB: ALK4
WCL

Supplement: Supplemental Figure 1 — Toxoplasma Induces ALK4/ActRII Dimerization and Activation. ActRII was immunoprecipitated from lysates prepared from mock- or parasite-infected cells (30′ post-infection) using anti ActRII or IgG as a control). Immune complexes were Western blotted to detect ALK4. ALK4 levels were assessed in whole cell lysates (WCL). [file Presentation_1.PPTX]
